# Phenomes: the current frontier in animal breeding

**DOI:** 10.1186/s12711-021-00618-1

**Published:** 2021-03-05

**Authors:** Miguel Pérez-Enciso, Juan P. Steibel

**Affiliations:** 1 ICREA, Passeig de Lluís Companys 23, 08010 Barcelona, Spain; 2 Centre for Research in Agricultural Genomics (CRAG), CSIC-IRTA-UAB-UB, Bellaterra, 08193 Barcelona, Spain; 3 Department of Animal Science, Michigan State University, East Lansing, MI 48824 USA; 4 Department of Fisheries and Wildlife, Michigan State University, East Lansing, MI 48824 USA

## Abstract

Improvements in genomic technologies have outpaced the most optimistic predictions, allowing industry-scale application of genomic selection. However, only marginal gains in genetic prediction accuracy can now be expected by increasing marker density up to sequence, unless causative mutations are identified. We argue that some of the most scientifically disrupting and industry-relevant challenges relate to ‘phenomics’ instead of ‘genomics’. Thanks to developments in sensor technology and artificial intelligence, there is a wide range of analytical tools that are already available and many more will be developed. We can now address some of the pressing societal demands on the industry, such as animal welfare concerns or efficiency in the use of resources. From the statistical and computational point of view, phenomics raises two important issues that require further work: penalization and dimension reduction. This will be complicated by the inherent heterogeneity and ‘missingness’ of the data. Overall, we can expect that precision livestock technologies will make it possible to collect hundreds of traits on a continuous basis from large numbers of animals. Perhaps the main revolution will come from redesigning animal breeding schemes to explicitly allow for high-dimensional phenomics. In the meantime, phenomics data will definitely enlighten our knowledge on the biological basis of phenotypes.

## Background

For the last two decades, genotyping and sequencing technologies have outpaced the most optimistic predictions, allowing industry-scale application of genomic selection. Today, genomics is a mature technology but unfortunately the momentum may be fading away. Increasing marker density follows the law of diminishing returns, unless the causative mutations are identified. Simulation and empirical results have shown that only marginal gains in genetic prediction accuracy can be expected by genome sequencing data instead of high-density genotyping data [1, 2]. This small advantage will probably vanish when the extra cost of computer storage and power that sequence analyses require compared to genotyping arrays are accounted for.

Looking forward, we argue that some of the most scientifically disrupting and industry-relevant challenges relate to ‘phenomics’ instead of ‘genomics’, as introduced in the premonitory words of Mike Coffey at the 2011 ICAR meeting, “In the age of the genotype, phenotype is king”. Phenomics, which is defined as ‘the acquisition of high-dimensional phenotypic data on an organism-wide scale’ [3], has flourished thanks to the development of all kinds of electronic devices and internet availability. Currently, sensors are able to inexpensively record images, videos, sounds, or a multitude of environmental parameters, making large-scale, continuous phenotyping possible. Improvements in this vast area occur at breath-taking pace.

However, there are some caveats in the ‘phenome’ concept [4]. While the genome, i.e., the DNA sequence, is finite and can in principle be fully characterized, the phenome is not a closed, fully-defined entity, and it will never be. An infinite number of phenotypes can be imagined: simply consider all the mathematical combinations of measured traits that can be defined. Furthermore, phenotypic measurements may involve several individuals simultaneously, as in many welfare and behavioral traits. Therefore, a phenome will always be a subset of an infinite number of possible measurements that may span several individuals. The difference with a ‘standard’ breeding setting is that, in the new paradigm, both the genome and the phenome are high-dimensional variables. Furthermore, whereas genome data are relatively homogeneous, phenome measurements can be highly heterogeneous and time-dependent. An example is the composition of microbiota, which change from birth to adult stages and may vary with health status. In this sense, it is important to realize that phenomics ‘big data’ problems arise because of the heterogeneity and rapid change over time of the data, not because of their size.

Compared to plant breeding or human genetics [3], animal phenomics has received somewhat less attention. This is surprising since new technologies allow the assessment of new phenotypes that are in high demand by the society, such as those related to animal welfare, resilience, disease incidence or resource use efficiency [5–8]. Fortunately, recent work indicates that animal phenomics is becoming popular in the animal sciences too, e.g., [9–12], and as reflected in public initiatives such as the USDA Agricultural Genomes to Phenomes Initiative AG2PI (https://www.ag2pi.org/).

The novelty around high-throughput phenotyping in farm animal populations comes from two angles: (1) novel traits can be defined and measured that could not be recorded before, and (2) classical traits can now be observed on an almost continuous basis and in a non-invasive way on large numbers of animals under normal production environments. However, we can expect the datasets to be partially incomplete, noisy and partially redundant, especially when traits are recorded on a continuous basis.

With this opinion paper, our aim is to foster discussion and further research in the area. Towards this, we briefly recall some of the most relevant traits that can be captured by modern technologies, and then discuss the future needs in terms of methods and algorithms and the possible long-term impact of phenomics in breeding. The focus of this note is on the use of sensors to collect measures on animals themselves, but note that sensors can and are used to record all sorts of environmental variables (climate, pathogen exposure, etc.), which are also highly relevant for animal management and breeding.

### Standard and new traits (re)visited

#### Behavior at the individual and group levels

Behavior and social interactions between animals can greatly affect production and performance phenotypes and are a major component of animal welfare. Given the difficulty of measuring behavior before the ‘phenomics era’, in genetic evaluations the effect of behavior on production traits has been either ignored or accounted for indirectly, e.g., by using social genetic effects models [13]. However, selection to modify behavior is possible since some behaviors, especially those related to aggression, are partially heritable [14]. Understanding and modifying the genetic factors that induce fighting between pairs of animals is valuable not only for selection, but also for management purposes, as groups could be formed based on the genetic makeup of animals that are expected to fight less with each other. As a result, welfare and productivity should increase.

**Fig. 1:**
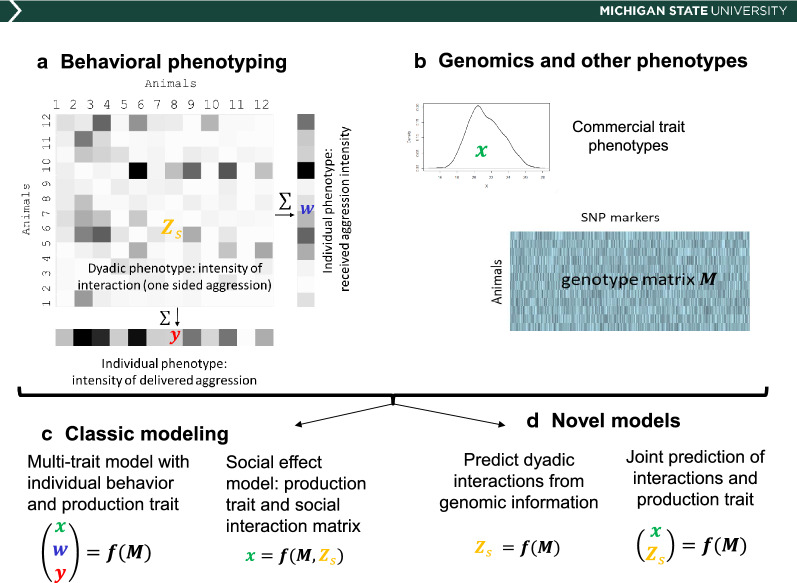
Emerging behavioral data in the phenomics era and the need for new models. **a** Behavioral phenotyping for social interactions results in a matrix of dyadic interactions, Zs, that can be collapsed to individual behavioral data (w and y). **b** Existing genomics and phenomics data can be integrated with behavioral phenotypes. **c** Classic genomic evaluation models focus on multi-trait analyses of individual behaviors or on social genetic effects models where the interaction matrix is used as a predictor of existing phenotypes. **d** In novel models, multi-trait analyses have to include full behavioral matrices to be able to predict the dyadic interactions from rker data

Today, behavioral traits can be measured with wearable sensors and computer vision techniques [15–17]. Behavior metrics can affect single individuals but often involve pairs or larger groups of animals. If behaviors are measured at the individual level, multi-trait direct genetic effects models can be used to obtain breeding values for the behaviors of interest together with production and welfare traits [18]. A less explored option is the modeling of behavior at the dyadic level (i.e., in individual pairs). This type of data has been used to parameterize social genetic effects models [19], but it could also be analyzed as a response matrix in a quantitative genetic study to determine, e.g., which genetic factors affect a group mate to be attacked. This type of analysis has not been performed yet, but dyadic behavioral data are being collected in livestock species and have been used to build social networks [20]. A typical interaction behavior is aggression (e.g., post-mixing attacks), where we distinguish between an individual measure and a group or dyadic measures. For instance, the total amount of time that each animal spends engaged in fighting represents an individual-level observation. But when the amount of time spent fighting is annotated for each pair of animals in a social group, a dyadic phenotypic dataset is generated. This type of datasets allows the development of specific, new modeling strategies (Fig. 1).

#### Gas emissions

Genetic selection for reduced greenhouse gas emissions is possible, given that the trait is heritable and genetically correlated with other traits such as milk production and residual feed intake [21]. Traditional measuring methods that involve respiration chambers and analysis of tracer elements are not readily scalable for application in high-throughput phenotyping. However, recently developed spectroscopy technology for “breath analysis” can be applied on a large scale to measure methane emissions. This technology can be incorporated into on-farm devices such as feeding kiosks or milking robots and used to measure instantaneous methane emissions on thousands of animals as they voluntarily approach measuring stations several times per day [22].

Like other phenotyping data described in this paper, incorporating high-throughput measures of emissions into genetic evaluations will require the integration of heterogeneous data sources. In this case, the heterogeneity will arise not only from a variety of measuring devices, but also from the different sampling schemes and measures. Highly processed and distilled data such as estimated total emissions per day will be available for some individuals together with raw instantaneous measurements of emission for other animals.

#### Feed efficiency

Feed efficiency largely determines farm exploitation costs and has been measured on farms on a limited scale for many years now. As with behavior, measuring feed efficiency accurately and massively has only been possible due to sensor technology, via automatic feed recording devices [23]. An inherent characteristic of feed intake and feeding behavior data obtained with automatic feeders is its incompleteness and its heterogeneity [24]. Because different devices are used to record and pre-process feed intake data, the measured traits may be slightly different. Sometimes, due to malfunction, there may be partially missing data, for instance, meal is recorded but no animal identity (ID) is attached to it or an animal’s visit is recorded but the feed intake is clearly wrongly measured or not measured at all. These peculiarities of the data recording process need to be accounted for in the analysis pipelines, including raw data processing, cleaning, and imputation.

On the data modeling side, there are opportunities to extract extra information and novel traits from automatic feeding station data. The sequences of the visits to the feeders, the time between visits and the co-occurrence of animals in multi-space feeders can be mined to uncover interactions between animals. Also, meal characteristics extracted from automatic feeders have been used to parameterize social genetic effects models [25]. Combining automatic feeding data with computer vision algorithms will likely shed further light on these problems.

#### Traditional phenotypes revisited

Sensor technology allows the measurement of new phenotypes but is also disrupting how ‘standard’ traits, such as weight or conformation, are recorded. In the case of weight and condition score, traditional methods typically require moving the animals and are labor intensive; for that reason, only a limited number of measures can be taken in each production cycle. With the advent of phenomics, these traditional phenotypes can be collected automatically on a continuous basis on a massive number of animals, without the need to disturb them. For instance, weight can be accurately measured using 3D imaging [17], or conformation features can be measured from images [26]. These precision livestock farming devices record under normal production conditions of market animals, and not only on elite animals in nucleus farms. Together with these measures, environmental records of similar temporal and spatial resolution can be obtained from weather stations or from barn environment controlling devices.

Nevertheless, the incorporation of automatically-measured body condition score or weight gain into existing genomic evaluations imply unique challenges. Data streams from continuously-measured live-weight on millions of animals will have to be summarized and cleaned before feeding them into existing genetic evaluations. Cleaning data by detecting outliers before fitting growth curves to individual records may not be efficient in the phenomics era; instead, robust data analysis techniques such as non-linear quantile regression can be used for growth curves using all available data points but avoiding the effect of outlying observations. Finally, having full growth curves from each animal will allow the evaluation of new traits around these traditional phenotypes.

#### Traceability and identification

Environmental data collected by sensors allow the study of genotype-by-environment interactions at a higher resolution scale than has been so far possible and the incorporation of production farm records into the evaluation of elite animals. However, for such uses, the data need to be linked in some way: phenotypic records of production animals need to be linked to parental genotypes and to environmental and production records. In symmetry, continuous individual identification throughout the production cycle will open the opportunity to record many new traits. Thus, the needs for animal identification and tracking as well as the synchronization of real-time data streams will increase.

Currently, individual identification is attained through computer vision of marked or unmarked animals or using wearable radio frequency identification (RFID) devices that remain with each animal for the duration of their productive life. Computer vision algorithms for animal identification use different techniques, such as natural variation in appearance (conformation, coat color, etc.) of the whole animal, visual marking or tags that are permanently or temporarily attached to the animals [27–29]. Ideally, a computer vision algorithm will work using images of panoramic views of the animal space (pen or barn), which are taken with ceiling or high-wall mounted cameras that include images of several animals in the same picture frame.

Automatic, reliable animal identification is not a solved issue. Among the challenges that need to be addressed are: (1) improving current computer vision algorithms for animal ID and tracking using top-view cameras in normal production conditions, (2) the integration and synchronization of multiple data streams (e.g., RFID detection logs with video streams from more than one video camera), and (3) the ascertainment of animal identification or accounting for uncertain ID. For instance, what is to be done if the animal identification algorithm produces two likely ID with a roughly similar probability for a single animal image? Shall the ID be treated as missing data? Or should the uncertainty be propagated into the animal genetic evaluation by using methods similar to those proposed for dealing with uncertain paternity [30–32]? These questions will be revisited when these kinds of data streams become common.

### High dimensionality

At the end of the day, phenomics technologies deliver highly-dimensional data that need to be processed and incorporated into breeding and management decisions. Two main, related statistical issues are relevant in this context: dimension reduction and penalization. Dimension reduction consists of obtaining new ‘synthetic’ variables that are combinations of the original dimensions. A usual justification of dimension reduction is that only a few dimensions are actually relevant, and that dimensionality is artificially high. Penalization refers to setting constraints of the parameter solutions in the predictive model.

**Fig. 2:**
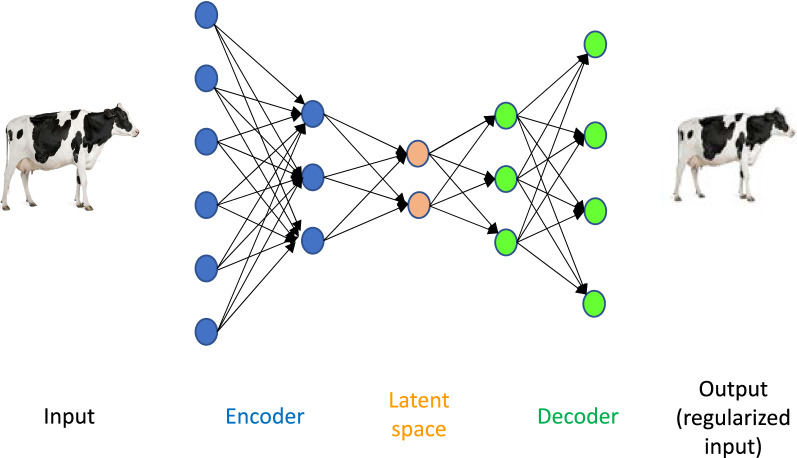
Representation of an Autoencoder. Autoencoders (AE) are deep neural networks where the input and output are the same (in this case, multi-channel pixel intensity values from images of livestock). They consist of an encoder that codes the input in a low dimensional latent space and a decoder that transforms back the input into a regularized version. Variational autoencoders (VAE) generate a probability function instead of a point latent space. Then, random numbers are drawn and transformed into simulated images by the decoder. Applications of AE and VAE to phenomics remain to be explored, but they can be used for unsupervised learning and imputation. The figure of the cow is from www.dreamstime.com

In practice, dimension reduction techniques are mainly used for visualization. New variables in the reduced dimensional space are derived to retain the original data pattern as faithfully as possible. In principal component analysis (PCA), the new variables are the linear combinations of the original phenotypes that explain the maximum possible variance, with the additional constraint of being orthogonal between them. PCA, together with multidimensional scaling (MDS), are perhaps the most widely used dimension reduction tools, but interesting and less well-known options exist and can be good alternatives. Some of these provide nonlinear approximations, in contrast to linear PCA. An ‘autoencoder’ (AE) is one of such nonlinear alternatives [33]. Autoencoders are ‘deep learning’ (DL) algorithms, i.e., they are based on several stacked layers of neurons (Fig. 2). However, in contrast to a typical DL network, where output and input are different, both input and output are the same in AE. Thus, they are unsupervised techniques, as they are mainly dimensionality reduction methods. If no restriction is set, the optimum AE solution is the identity vector and reconstructed output is identical to input. It is then necessary to set some constraints, i.e., penalizations, to optimize the AE network. In the specific case of one single layer and a linear activation function, it has been shown that AE and PCA yield basically the same solution [34].

**Fig. 3:**
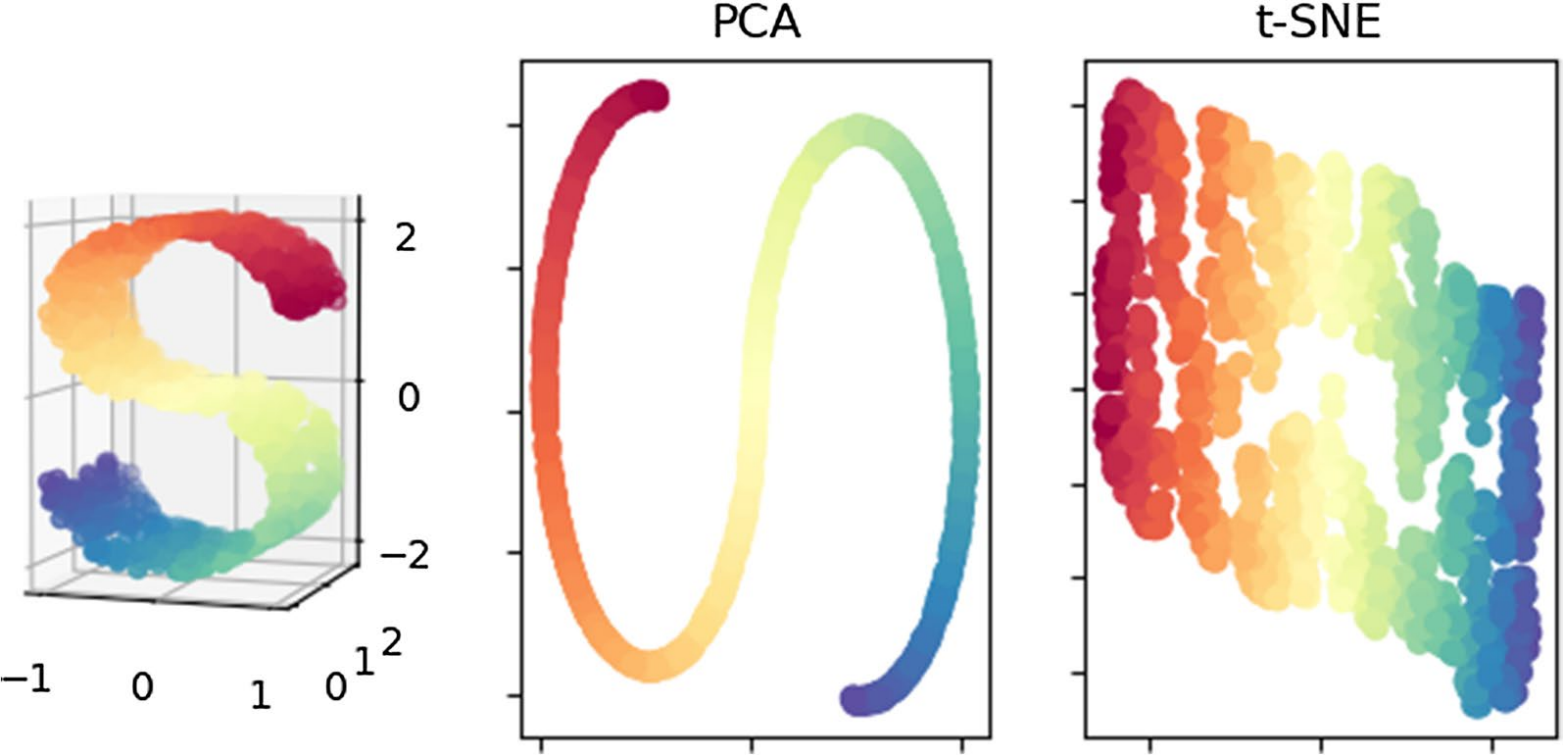
Comparison between PCA and t-SNE dimensional reduction methods using the 3D ‘S’ shape in left panel. Note that PCA is a projection that aims at maintaining the maximum variance, whereas t-SNE keeps local similarity in the low dimensional space. For instance, PCA projection clearly maintains the original ‘S’ shape, where the third dimension is lost. In contrast, the plot produced by t-SNE is better at representing local relative distances, where the first dimension reproduces the contour of the shape and the second dimension, the relative position within that part of the contour. As a result of different targets, very different pictures emerge. Implications for phenomics are to be explored. Plot using scikit [53], slightly modified from J. Vanderplas code (https://scikit-learn.org/stable/auto_examples/manifold/plot_compare_methods.html)

Another interesting dimension reduction algorithm is t-distributed stochastic neighbor embedding (t-SNE, van der Maaten and Hinton [35]). The goal of t-SNE is to find a low dimensional representation whereby similar data-points in the original space are shown together and distant samples are shown far apart. The most important difference between PCA and t-SNE is that the former is a projection onto a lower dimensional space, whereas the latter is a representation strategy. Besides, by construction, PCA is aimed at maximizing distances when samples are plotted in the low dimensional space. MDS is a generalized approach for PCA and, similar to t-SNE, it is designed for preserving distances between samples. However, t-SNE offers a series of advantages over MDS; in particular, it reduces the tendency of samples to cluster together, which is caused by a large number of dimensions and results in increased resolution. It seems that neither t-SNE nor autoencoders are popular in animal phenomics, but they are techniques worth exploring since they offer complementary information to standard linear methods. Figure 3 illustrates how different algorithms can provide dramatically different representations in the low dimensional space.

Penalization refers to setting constraints on variables to avoid collinearity and overfitting problems when the number of variables is large. As well known, penalization is needed to avoid the method ‘learning’ irreproducible noise, i.e., one of the ‘curses of dimensionality’ [36]. Two main types of regularization have been proposed: L1 and L2. L1 consists in setting a constraint on the sum of absolute values of the solutions, whereas L2 refers to the sum of squared solutions [37]. Concepts such as prior information in a Bayesian framework are equivalent to penalization. Although numerous methods with different names have been proposed in the Bayesian literature, most of them can be unified by realizing that they simply differ in the prior chosen [38]. In addition, deep learning technologies have developed specific additional regularization strategies. One of them is called ‘dropout’, which consists of randomly removing ‘neurons’ from the inner layers to force the system to use fewer parameters. In spite of its distinct definition, dropout can be interpreted from a Bayesian point of view, and thus can be viewed within the usual penalization framework [39]. Another approach used in some DL models is direct L1 or L2 penalization on the neuron weights, i.e., a constraint is added on the sum of the absolute or square value of the weights.

Complexity, heterogeneity, and especially data size will notably increase in the phenomics era, which will have repercussions in the modeling approaches. It is sometimes erroneously believed that the influence of the prior vanishes with large datasets, but this is not the case since the prior will always have an effect on the solution, regardless of the amount of data [38]. Thus, it is worth studying the impact of alternative regularization strategies, as we cannot expect that a single strategy will be optimum—from the predictive performance point of view—in all cases.

As just mentioned, the term ‘curse of dimensionality’ has become popular in statistics and by extension in breeding to mean that increasing ‘unnecessarily’ the complexity of a model leads to poor predictive performance [37]. The term ‘unnecessarily’ can be read as ‘without penalization’. According to Donoho [36], the term was initially coined to reflect the impossibility of enumerating all possible models as the number of variables grows. However, high dimensionality is a ‘blessing’ for many purposes, an aspect that is less widely acknowledged in our field. One reason is that having many highly correlated variables helps to smooth out noise. A further, more relevant advantage is that increasing the number of variables usually results in penalized models with improved predictive performance [40]. Finally, increasing dimensionality allows a gain in biological insight.

### The way ahead

Strangely, one of the first obstacles that will need to be solved for routine phenome collection is access to broadband internet. Even in the USA, as much as 40% rural farms lack reliable access to broadband [9]. Aside from infrastructure issues, here we wish to focus on methodological issues.

Quality control and visualization techniques should be a first step in a phenomics pipeline. As phenotyping becomes a large-scale objective, reliability can be compromised and heterogeneity in environmental conditions may increase. However, bias can be a much more serious danger than error because phenome data will not be collected randomly. Likely, special attention will be paid to elite farms or breeding nucleus, and certain phenotypes will be collected preferentially on specific farms. We can also expect differences in accuracy for data from elite farms compared to data from commercial farms, which will have to be accounted for through proper modeling.

As a result, phenome data will also be highly unbalanced: the kind and amount of data available will vary largely across individuals, even if sensors are widely spread and collect information routinely. It is unlikely that identical phenotypes are recorded on different farms or in different periods, whether milk recording or behavior measurements. This can be an important obstacle, since it will require either removal of samples and/or imputation of missing values. We need to develop efficient and accurate imputation tools or use methods dealing with missing data directly. In this aspect, plant phenomics measurements can often be more systematically compared and measured on a larger scale than in livestock.

**Fig. 4:**
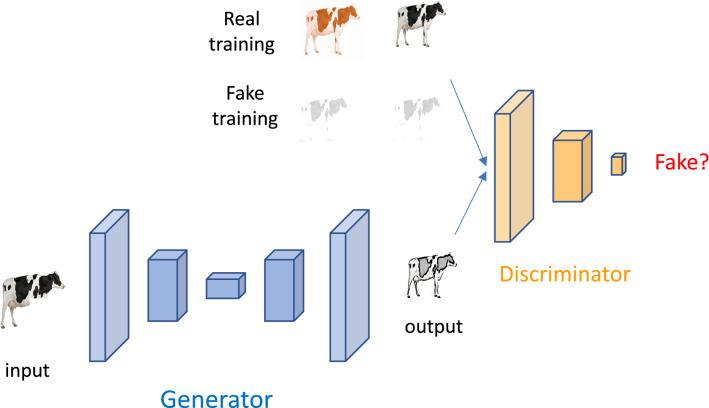
Representation of a generative adversarial network (GAN). This GAN aims at filling holes in an image (‘inpainting’, i.e., imputation). The generator network simulates a new image out of the input picture of the cow, which does not have front legs. The discriminator is trained with images that are true or fake and learns how a real cow looks like. The generator discriminates whether the generated image is true or fake, the result is passed on to the generator so that it can improve the quality of the output image. Each of the rectangles in the generator and the discriminator represent a group of neuron layers as in Fig. 3, the shape is approximately proportional to its dimension. The figure of the cow is from www.dreamstime.com

Given that missing data is unavoidable, imputation will be needed. This is a wide area and numerous approaches exist depending on the specific problem, e.g., [41, 42]. However, it should be noted that most imputation techniques assume that missing data is at random, a condition that phenome data will unlikely fulfill, as discussed above. A further issue with phenomics data is their heterogeneity and thus no general imputation rule can be given. In addition to standard imputation techniques [42], alternative approaches exist based on deep learning that, to our knowledge, have not been used in this area and can be promising. For instance, autoencoders can be used to fill ‘holes’ in data, in particular those with a spatial pattern such as image and video. The standard autoencoders output is a regularized representation of the original input. This is accomplished via an ‘encoder’ that transforms the data into a ‘latent space’ and a ‘decoder’ that takes the latent space coordinates and outputs a regularized image. Instead of recoding the input into latent space coordinates, variational autoencoders (VAE) generate a probabilistic function to describe an observation in the latent space. As a result, realistic data-points can be generated. For instance, VAE have been used to increase the resolution of pictures or to restore damaged pictures, a problem that is conceptually identical to imputation. In the same vein, generational adversarial networks (GAN) are DL algorithms that can reproduce high-dimensional variables. So far, GAN have been mostly used to generate images, e.g., to generate very high-resolution pictures out of incomplete or low-resolution images, or even videos [43, 44]. Figure 4 represents a scheme of a GAN for ‘inpainting’, i.e., completing missing parts in an image. Compared to VAE, GAN are much more flexible, yet they are more difficult and slower to train. They also require larger amounts of data. Applying GAN and VAE concepts to imputation in phenomics is a promising area of research, given their flexibility and lack of distributional assumptions. However, caution applies, since these methods have been tested mainly with image data, and performance in other scenarios has not been much explored.

However, even with complete data of reasonable size, there are important gaps in the current methodology and software that should be filled. There is an enormous range of analytical tools to be developed. Automatic individual identification allowing for free range, or at least movement in group-housed animals under confinement is needed. This can be accomplished, e.g., using continuous video recording and tracking. Algorithms that automatically extract phenotypes from image, video, sound records are a very active area of research. Standard metrics (e.g., Hausdorff, Euclidean, etc.) to measure similarities between images should be adapted to livestock, and new metrics for videos should be implemented as they are required to compare animal behaviors. Automatic conformation measures [26] must be improved to be performed in vivo with minimal human intervention. A further challenge is to incorporate all this information into genome predictions.

Not all species and breeding programs will benefit equally from phenomics. For instance, the aquaculture industry is highly advanced technologically and many measurements are difficult to obtain manually. Here, the use of phenomic technologies is far more widespread than in other species. Small ruminants in extensive farming may represent the opposite extreme. Yet, sensor technology can provide accurate animal tracking and remote measurement of physiological and environmental variables outdoors, which could boost productivity and health precisely in traditional, low-input agriculture conditions. In general, those production systems where precision agriculture and precision livestock management are being implemented will be better equipped to collect relevant phenomics data, and the limitation in those cases will be on data storage and transmission as well as data labeling and individual identification.

Although model interpretability is an issue [45, 46], experience shows that opening the ‘black box’ is not needed for accurate prediction [40]. Nevertheless, phenomics data will definitely enlighten the biological basis of phenotypes and will complement genotype data. As geneticists, we are often in the quest of DNA causative polymorphisms. However, focusing on this search may often impede the detection of non-genetic factors that have a stronger effect on the phenotypic expression than the causative mutations. This has been observed, e.g., in expression quantitative trait loci (QTL) studies [47]. We contend that large-scale phenotyping is important per se, independently of whether genome data are available or not. In this context, structural equation modeling [48] but also unsupervised learning gains relevance. Influential DL pioneers, such as Yann LeCun, have actually argued that the future of artificial intelligence lies in unsupervised learning (https://www.youtube.com/watch?v=Ount2Y4qxQo&t=1072s, NIPS conference, 2016). This is because unlabeled data (unsuited for supervised learning) are far more abundant than labeled data and, more importantly, because unsupervised learning resembles more precisely how the human brain actually works. As phenome data are collected over the years in the same or similar breeding schemes, by using unsupervised learning methods, we will gain invaluable knowledge on the effects of selection on the whole organism. For instance, unsupervised learning may uncover unsuspected relationships between traits or between traits and environmental variables. We could, e.g., discover how some selection barriers can be overcome or how to optimize economic weights dynamically.

Phenomics is a hot, promising area but is not exempt from risks and cannot be considered a panacea. As Cole et al. [12] warn us, ‘these new approaches have their own challenges, ranging from bias to interpretability and there is a temptation to oversell outcomes’. One serious issue is that, in contrast to genotypes, phenome data may not be easily transferable or comparable across farms. Often, measurement technology is proprietary and several systems, e.g., to measure methane emissions, coexist. Algorithms to transform raw sensor data into meaningful measurements and sensors themselves rapidly change over time, making it difficult to analyze data longitudinally. Standards and open-source algorithms in the sensor industry are necessary to fully unravel the potential of phenomics. A way to foster the development of novel algorithms in this field is the distribution of relevant datasets to the research community and the organization of activities (hackathons, competitions and journal special issues) around the analysis of such datasets. This is being done successfully in the fields of autonomous vehicles, computer vision [49] and in many other areas (e.g., https://www.kaggle.com/competitions).

Perhaps the main revolution will come from redesigning animal breeding schemes to explicitly allow for highly dimensional phenomics. To begin with, do we need new definitions of breeding values? Genotype × environment (G × E) interactions can be an inspiring concept here. In essence, including G × E interactions in the model is equivalent to providing a function for the breeding value instead of a single value, a reaction norm. In the phenomics era, traditional point breeding values might be replaced by high-dimensional generative functions. It is not clear at this point how this will be accomplished. A natural approach is to use phenomics data to integrate mechanistic biological models into genetic evaluation. Examples are growth crop models in plants such as those developed by Totir et al. [50, 51]. More generally, we hypothesize that phenomics-based genomic evaluations will likely be a combination of standard statistics methods with generative machine-learning and simulation tools. In a recent work, de los Campos et al. [52] applied large-scale simulation conditioned on genotype and environmental variables to predict future performance but, instead of a point prediction, a whole distribution over uncertain, future climate conditions was generated. We can imagine that future phenomics-assisted breeding schemes will be able to simulate expected complex phenotypes under a range of potential environmental conditions for each target genotype.

## Conclusions

The influence of phenomics in livestock breeding has only begun and much work remains to be done. High dimensionality is a ‘blessing’ rather than a ‘curse’ to improve prediction [40] and we should not be afraid of it. High dimensionality should also help breeders to fine tune which are the most relevant phenotypes, and what are the expected constraints. Making progress in phenomics depends on the fast-developing field of sensor technology and machine learning. This reinforces the idea that the breeders of the future will require sound agronomic and biological backgrounds as well as a solid training in statistical and machine learning. This may seem paradoxical, but it will be the case because easy-to-use, powerful programming libraries and code will be widely available, whereas interpretation of results and application in breeding schemes require specific biological and agronomic knowledge. Last but not least, breeders trained in phenomics who can effectively collaborate with biologists, producers, engineers and computer scientists will have increased chances of succeeding in the job market.
